# Traditional Chinese Medicine Constitution Correlated with Ischemic Stroke: A Systematic Review and Meta-Analysis

**DOI:** 10.1155/2021/5524925

**Published:** 2021-06-24

**Authors:** Tianyi Zhang, Hui Luo, Dawei Wei, Xiaolong Xie, Cinyu Yang, Bowen Liu, Ying Gao

**Affiliations:** ^1^Dongzhimen Hospital, Beijing University of Chinese Medicine, 5 Haiyuncang, Dongcheng District, Beijing 100700, China; ^2^Institute for Tibetan Medicine, China Tibetology Research Center, Beijing 100101, China

## Abstract

**Objective:**

To investigate the correlation between traditional Chinese medicine (TCM) body constitution and ischemic stroke (IS).

**Methods:**

Literature search was conducted in databases including Wanfang database, Chongqing VIP, China National Knowledge Infrastructure, Embase, and PubMed from inception to November 16, 2020. Observational studies evaluating the association between TCM body constitution and IS were included for analysis. The distribution of body constitutional types in IS patients was pooled into meta-analysis. The correlation between constitution and IS was presented by the odds ratio (OR) and 95% CI through the comparison between IS and the general population.

**Results:**

41 studies involving 11,211 participants were included. Among the nine constitution types, qi-deficiency constitution (QDC), phlegm-dampness constitution (PDC), and blood stasis constitution (BSC) are the common types, accounting for 25% [0.22, 0.29], 23% [0.20, 0.29], and 17% [0.13, 0.22], respectively, in IS patients. The proportion of PDC and QDC among IS patients is 2.34 times and 3.47 times higher than that in the general population, respectively (OR and 95% CI: 2.34 [1.39, 3.94], 3.47 [1.61, 7.50], respectively).

**Conclusion:**

PDC and QDC are the common constitutions in IS patients and may have a potential correlation with the incidence of IS. Due to the low or moderate quality of included studies, more well-designed prospective studies are warranted to further evaluate the relationship between TCM constitutions and IS.

## 1. Introduction

Stroke is the second most mortal disease globally. Ischemic stroke, a sudden functional loss caused by the cut-off of the bloodstream to the brain, is the most common type that accounts for over 85% of all cases [1]. Every year, about 6 million people die from stroke globally, and even worse, the illness, disability, and premature death caused by stroke will double by 2035 [2]. The recurrence rate within a decade is as high as 39.2% [3–5]. On the one hand, the treatment and rehabilitation of stroke will bring huge economic burden to the society, but on the other hand the current therapeutic approaches are insufficient for reducing deaths caused by stroke. Prevention of stroke is of great significance to the family and the society. Risk factor management is important for preventing stroke. In studies focusing on stroke risk factors, the Framingham Stroke Scale is currently the most widely used stroke risk assessment tool in western countries [6]. However, it overestimated the risk of stroke in Chinese [7]. Besides, these studies focus on defining risk factors of stroke, without providing a clear definition of the high-risk group of stroke [8].

In traditional Chinese medicine, preventative treatment before the disease onset has been practiced for thousands of years. TCM theory states that Chinese medicine has a great advantage in the preventive treatment of disease. TCM constitution identification is the effective approach of “treating disease before onset.” [9] The TCM constitution is a relatively stable and integrated intrinsic feature of physiological function, morphological structure, and psychological state formed by innate endowment and acquired disposition. The TCM constitution can be classified into nine basic types: yang-deficiency constitution (YADC), qi-deficiency constitution (QDC), qi stagnation constitution (QSC), phlegm-dampness constitution (PDC), yin-deficiency constitution (YIDC), damp-heat constitution (DHC), blood stasis constitution (BSC), balance constitution (BC), and inherited special constitution (ISC). Among these, BC is normal constitution and all the others are biased constitutions. People with different constitution have different characteristics in physiological manifestation, pathological state and morbidity tendency, and so on. For instance, the BC, formed by the harmony of yin, yang, qi, and blood, is characterized by ruddy complexion, vigorous energy, and moderate posture. People with BC can adapt to the environment easily and are unlikely to suffer from diseases.

There are emerging clinical studies focusing on TCM constitution and its correlation with IS. These studies provide a basis for investigating the clinical distribution of TCM constitution in IS patients. However, no systematic review is found among existing studies. Thus, a summary is needed to incorporate the existing literature. To provide solid evidence for clinical and scientific research based on larger sample, a meta-analysis is conducted on the above studies.

## 2. Methods

### 2.1. Registration

The protocol was registered and published at PROSPERO (CRD42020209602), accessible at https://www.crd.york.ac.uk/PROSPERO/display_record.php?RecordID=209602.

### 2.2. Searching Strategy

#### 2.2.1. Literature Search

We collected clinical literatures on the association between TCM constitution and IS from databases such as Wanfang Database, Chongqing VIP, China National Knowledge Infrastructure, Embase, and PubMed. The literature is either in English or in Chinese. The dates range from the day when the database was founded to November 16, 2020. The keywords are “ischemic stroke,” “constitution,” and “Chinese medicine.” For Chinese databases, searching strategies are “ischemic stroke + constitution” in title or abstract, and “Chinese medicine” in full text. For English databases, search strategies are ((ischemic stroke) AND constitution AND (TCM OR Chinese medicine)).

### 2.3. Eligibility Criteria

#### 2.3.1. Inclusion

(1) All clinical studies (including cross-sectional studies, case-control studies, and cohort studies) in Chinese or English on TCM constitution and ischemic stroke correlation are included. (2) All patients in studies should be clearly diagnosed with ischemic stroke. (3) TCM constitution recognition: TCM constitution is recognized by “classification and determination of TCM constitution” norm report by China Correlation of Chinese Medicine (CACM) in 2009 [10].

#### 2.3.2. Exclusion

(1) The basic characteristic of patients are not reported (gender, age etc.). (2) The results' data reported are deficient. 3. Research reports share the same patient samples.

### 2.4. Literature Screening and Data Extraction

All papers are first screened by title/abstract in line with the eligibility criteria on NoteExpress software. Those that pass the initial screen are checked throughout. Then, only those that pass the two screenings are used for data extraction. Each paper is screened and extracted by two investigators independently, and the results are cross-checked in every step. In the case of divergence in cross-check, the checked paper will be examined by the 3rd investigator for the final decision. The main extract data include researcher's name, study type, period and region, participant source, sample size, age, gender, constitution results, and measures of quality control.

### 2.5. Quality Evaluation

The Unit States Agency for Healthcare Research (AHRQ) publishes standards to evaluate cross-sectional study [11], including 11 items with the highest mark of 11. The items include observation period, eligibility criteria, research object continuity, data source, and quality control. As for the mark, 0–3 is considered as low quality, 4–7 as medium quality, and 8–11 as high quality. The Newcastle-Ottawa scale (NOS) recommends case-control study and cohort study [12], containing 11 items in 3 prospects. The NOS mainly evaluates the comparability between groups, population selection, and evaluation of exposure factors. Its highest mark is 9, and any mark over 6 is considered as high quality.

### 2.6. Statistical Analysis

Two meta-analysis sets are constructed based on the 41 studies included.

#### 2.6.1. Analysis Dataset 1

The analysis dataset 1 is a meta-analysis of the association between TCM constitution and IS. This part aims to explore the risk TCM constitution in IS. The meta-analysis is conducted on the studies that report distribution of TCM constitution in both IS patients and general population. ReviewManager (version 5.3) software is used for the meta-analysis. The effect values are described by odds ratio (OR) and 95% confidence interval (95%CI). *I*^2^ is defined as the heterogeneity of the meta-analysis. If the heterogeneity is low (*I*^2^ ≤ 25%), the fix effect model will be chosen; if the heterogeneity is high (25% < *I*^2^ < 75%), the random effect model will be chosen; if the heterogeneity is much higher (*I*^2^ ≥ 75%), qualitative description will replace meta-analysis.

#### 2.6.2. Analysis Dataset 2

The analysis dataset 2 is a meta-analysis of the distribution of TCM constitution in IS patients. This part aims to explore the total rate of different TCM constitution types in patients. The meta-analysis is conducted on the studies that report distribution of different constitution types in IS patients. The data of studies in analysis 2 is featured with that they only provide the number of people and events in one group. At present, ReviewManager, a commonly used meta-analysis software, cannot achieve single-rate meta-analysis. In this analysis, R version 3.2.2 is used for this meta-analysis. The results are shown by total ratio of different TCM constitution types in IS population and its 95% CI. When the heterogeneity is much higher, the subgroup analysis will be conducted according to region, gender, and related factors.

## 3. Results

### 3.1. Literature Search

**Figure 1:**
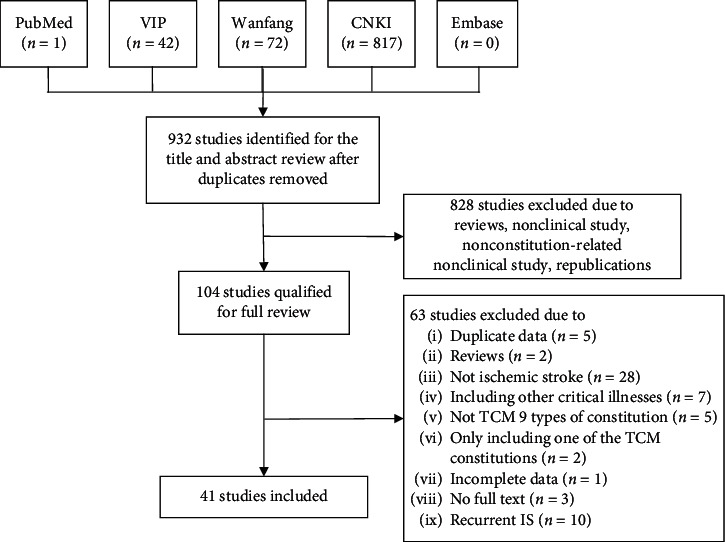
Flow diagram and results of literature searching.

Following the searching strategy, we collected a total of 932 papers, with 41 passing the eligibility criteria. The paper includes 11,211 eligible subjects. The flow diagram and results of literature searching are shown in Figure 1 [13–53]. One of the studies is reported in English and the rest are in Chinese.  Table 1 shows the fundamental features of the studies.

#### 3.1.1. Meta-Analysis 1: Meta-Analysis of the Distribution of TCM Constitution in IS and the General Population

**Figure 2:**
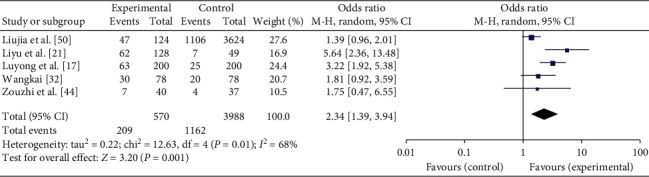
Meta-analysis of QDC's distribution in IS and the general population.

*QDC.* In total, 5 articles investigate the QDC's distribution in IS and the general population [17, 21, 32, 44, 50]. Heterogeneity is tested among the studies (*I*^2^ = 68%, *P*=.01), and the random-effects model is implemented. The meta-analysis reveals that OR = 2.34, 95% CI is [1.39, 3.94] and finds statistically significant differences (*P*=.001). The results are shown in Figure 2.

**Figure 3:**
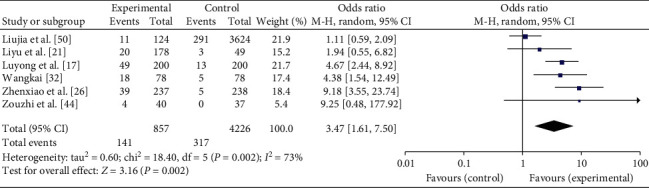
Meta-analysis of PDC's distribution in IS and the general population.

*PDC*. In total, 6 articles report PDC's distribution in IS and the general population [17, 21, 26, 32, 44, 50]. Heterogeneity is tested among the studies (*I*^*2*^ = 73%, *P*=.002) and random-effects model is used. Meta-analysis reveals that OR = 3.47, 95% CI [1.61, 7.50], indicating statistically significant differences (*P*=0.002). The results are shown in Figure 3.

*Other TCM Constitutions*. In total, 5 articles report blood stasis constitution's distribution in IS and the general population [18, 22, 33, 45, 51]. And 4 report yang-deficiency constitution (YADC), yin-deficiency constitution (YIDC), damp-heat constitution (DHC), qi stagnation constitution (QSC), balance constitution (BC), and inherited special constitution (ISC) [18, 22, 45, 51]. The meta-analysis reveals that the difference is not statistically significant (*P* > .05). The results are listed in Table 2.

#### 3.1.2. Meta-Analysis 2: Meta-Analysis of the Distribution of TCM Constitutions in IS Patients

The meta-analysis is conducted on each TCM constitution type of IS patients report as an index. Among the 41 studies, 1 case-control study of PDC only covers the proportion of PDC, and the other 40 studies report data on the proportion of various types of TCM constitution. Therefore, 41 studies are included in the meta-analysis of PDC. The results show that these studies are highly heterogenous. Considering the particularity of the cross-sectional study in the field of TCM constitution (see Section 4.3), the random effect model is used for meta-analysis. PDC, QDC, and blood stasis constitution (BSC) accounts for more than 15% of all. The results of meta-analysis are shown in a forest map, and the rest of the TCM constitutions less than 15% are listed in a table.

**Figure 4:**
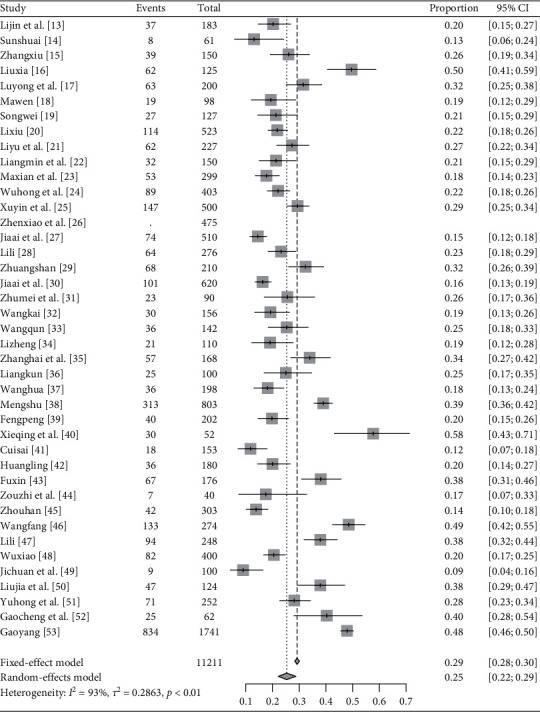
Meta-analysis of QDC's rate in IS patients.

*QDC*. In total, 40 articles report QDC's distribution in IS patients [13–25, 27–53]. Heterogeneity is tested among the studies (*I*^2^ = 93%, *P* < .01) and random-effects model is used. Meta-analysis finds the rate = 25% and 95% CI is [0.22, 0.29]. The results are shown in Figure 4.

**Figure 5:**
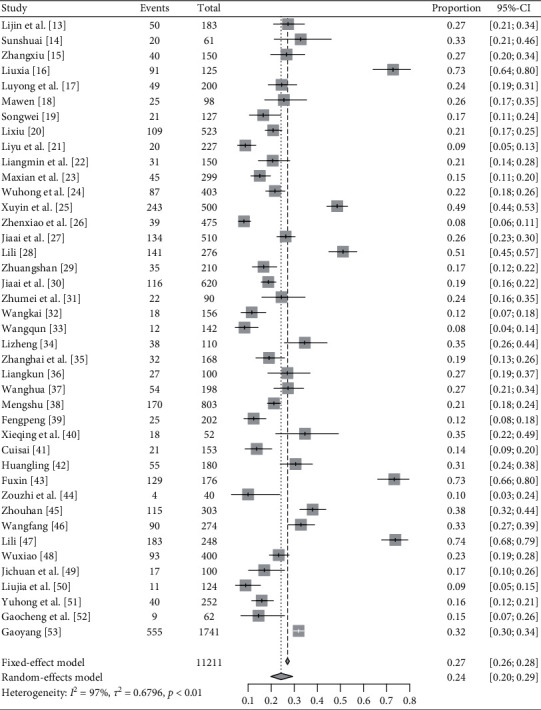
Meta-analysis of PDC's rate in IS patients.

*PDC*. In total, 41 articles report PDC's distribution in IS patients [13–53]. Heterogeneity is tested among all studies (*I*^2^ = 96%, *P*<0.01) and random-effects model is used. Meta-analysis finds that rate = 23% and 95% CI is [0.20, 0.29]. The results are shown in Figure 5.

**Figure 6:**
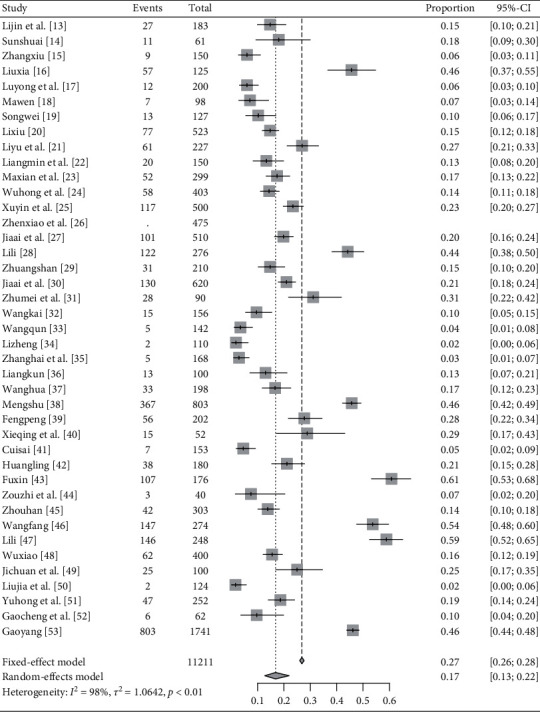
Meta-analysis of BSC's rate in IS patients.

*BSC*. In total, 40 articles report blood stasis constitution's distribution in IS patients [13–53]. Heterogeneity is tested out among the studies (*I*^2^ = 98%, *P*<0.01) and random-effects model is used. Meta-analysis finds that rate = 17% and 95% CI is [0.13, 0.22]. The results are shown in Figure 6.

*Other TCM Constitutions*. In total, 40 articles that report YADC, YIDC, DHC, QSC, BC, and ISC's distribution in IS patients [13–53]. Meta-analysis finds the rate is lower than 15%. The detailed results are shown in Table 3.

#### 3.1.3. Subgroup Analysis: Meta-Analysis of TCM Constitution's Distribution in Different Areas and Genders

*Different Areas.* In total, 7 articles report the distribution of constitution in IS patients in north China [14, 16, 21, 25, 37, 46, 48]. Meta-analysis is shown by rate [95% CI]. It finds PDC accounts for 33% [0.20–0.50], QDC for 28% [0.20–0.39], and BSC for 27% [0.18–0.38]. In total, 16 articles report the distribution of constitution in east China [15, 17–20, 23, 24, 26, 29, 33, 35, 36, 41, 42, 51]. It finds that PDC accounts for 18% [0.15–0.21], QDC for 25% [0.22–0.29], and BSC for 9% [0.06–0.13]. And 10 articles report the distribution of constitution in central south China [13, 23, 28, 31, 32, 34, 38, 44, 45, 49]. It finds that PDC accounts for 25% [0.18–0.33], QDC for 20% [0.16–0.26], and BSC for 17% [0.10–0.28]. A total of 5 articles report the distribution of constitution in northeast China [27, 30, 39, 43, 47]. It finds that PDC accounts for 38% [0.17–0.65], QDC for 24% [0.16–0.34], and BSC for 36% [0.21–0.53].

*Different Genders.* In total, 10 articles report the specific data of constitutions types by gender [15, 20, 21, 24, 25, 32, 37, 45, 48, 51]. No gender difference is found in the distribution of PDC in IS patients (OR: 1.09, 95% CI: 0.72–1.65 *P* = .68), in that of QDC (OR: 1.18, 95% CI: 0.77–1.81, *P*=0.46), and neither in that of BSC (OR: 0.78, 95% CI: 0.42–1.46, *P*=.43).

## 4. Discussion

### 4.1. Analysis of TCM Constitution Types in IS and the General Population

The meta-analysis based on 11,211 samples finds PDC, QDC, and BSC, respectively, account for (rate and 95% CI: 23% [0.20, 0.29], 25% [0.22, 0.29] and 17% [0.13, 0.22]) of all patients. The one based on 4,608 samples finds the proportion of PDC and QDC in IS patients is 2.34 and 3.47 times higher than that in the general population, respectively (OR and 95% CI: 2.34 [1.39, 3.94], 3.47 [1.61, 7.50]), with statistically significant differences. Based these findings, the study suggests PDC, QDC, and BSC are the main TCM constitution of IS patients, QDC and PDC constitution may be the risky factors of IS, and PDC is the most common one.

The distribution of TCM constitution types in IS patients is quite different from that in the general population. Professor Wang Qi conducted a cross-sectional field study on the distribution of TCM constitution, which includes 21,948 cases from 9 provinces and cities during 2005–2007 [54]. The results find that BC tops all, accounting for 32.14% among all. And QDC, DHC, and YADC are ranked in the top three in terms of biased constitutions, accounting for 13.42%, 9.08%, and 9.04%, respectively. In this meta-analysis, QDC has the highest proportion (25%) in all TCM constitution types, close to the proportion of BC in the general population, much higher than the proportion of QDC in the general population (13.42%). Similarly, PDC and BSC are like so. The BC only accounts for 7%. Therefore, it is advised to identify the constitution before the onset of the disease, and health education is an effective way to prevent the high-risk group from turning into bias constitution such as QDC, PDC, and BSC. In addition, identifying the bias constitution earlier could promote the return of bias constitution to BC through TCM therapies, which is of great significance to prevent the occurrence of IS.

In terms of the regional distribution proportion of TCM constitutions, the PDC and BSC occupy the highest proportions in Northeast China, followed by North China, Central South China, and East China. The QDC is most common in North China, followed by East China, Northeast China, and Central South China. The difference in distribution among regions is related to the climate, water, and dietary habit. TCM emphasizes the harmony between people and nature. So, this study may be closely related to climatic and dietary factors. The environment in North and Northeast China is colder and drier than the East and Central South China, and people in North and Northeast China prefer salty food and spirit. So, PDC and BSC have higher proportion there, while the climate in North and East China is warmer but not hot, moister but not humid. Besides, the food is light in these regions. So the proportion of QDC there is higher than that in the Northeast and the Central south China. Further studies need to be done to explore other reasons.

In terms of the proportion of gender distribution of TCM constitution, the proportion of PDC, QDC, and BSC is the same in males and females in this study. This is possibly because QDC, PDC, and BSC are the main constitution of IS, and the pathological mechanism results in the same constitution regardless of gender.

### 4.2. Methodological Problems in the Studies of TCM Constitution in IS Patients

This study discovers many methodological problems through quality evaluation. The international reporting standard for observational studies statement (Strengthening the Reporting of Observational Studies in Epidemiology, STROBE) has not been applied for format reports in any research included [55]. The reporting rate of each item is pretty low according to the evaluation standard of AHRQ cross-sectional study. Very few studies report the quality control measures clearly, which may lead to bias in the research results. The studies are highly heterogenous (I^2^ value is larger) from the meta-analysis of TCM constitution distribution.

From the perspective of research design, these studies do not contain enough basic information, including but not limited to the time range, the approach to including subjects, the methodological details of constitution identification, nationality, age, gender, body mass index, blood glucose, blood lipids, blood pressure, rejection rate, and funding status. In the statistical analysis of data, some studies omit the correlation between TCM constitutions and gender, age, disease degree, and other related factors. The individual studies find three or more types of mix constitution, or indicate the proportion of balanced constitution in the subjects is 0. This is because the researchers neglect that the TCM constitution scale should be based on the feelings of the past year, which leads to confusion on TCM constitution and TCM syndrome.

### 4.3. Limitations of the Study

This study analyzes the distribution proportion of different TCM constitutions in IS patients, and the correlation among region, gender, and TCM constitution. However, it fails to study the correlation between TCM constitution and nationality, occupation, age, marital status, course of disease, biochemical index, TCM syndrome, and other factors in IS patients, as it lacks data report in the original literature. This, it is more difficult to figure out the factors affecting TCM constitution.

The results are likely to be biased due to the high heterogeneity of the cross-section studies in the meta-analysis, which is related to many factors of each study such as the region, sample size, diagnostic criteria, and measurement methods. However, TCM constitution theory believes that the differences in constitution are due to region and gender. And the features of TCM constitution are reflected in the results of the national norm [54]. Hence, to describe the overall TCM constitution distribution features of IS patients in China, this study analyzes the data of original literature comprehensively and categorizes them by region and gender for more accurate results.

### 4.4. Enlightenment to Clinical and Scientific Research

The results suggest that the groups most in need of IS prevention are qi-deficiency, phlegm-dampness, and blood stasis constitution. Some effective prevention measures are health education, lifestyle, and TCM intervention. The TCM constitution could be applied for the clinical treatment of ischemic stroke (QDC, PHC, BSC, and other types). The differentiated treatment is offered based on the TCM constitution, and the conformational effect between TCM constitution and syndrome.

In this study, the evidence-based medicine research method is adopted in the field of TCM constitution in IS patients for the first time, and the literature on the relationship between IS and TCM constitution is systematically evaluated and analyzed by meta-analysis. The research ideas and methods can be used as a reference for the literature review on the association between diseases and TCM constitution in the future. It is suggested that a scientific and feasible research plan should be formulated and published before implementing “body-disease related” clinical studies (cross-sectional studies, case-control studies, and cohort studies). The process could improve the research plan quality though peer review. A general population control group could be organized for the cross-sectional study to compare the differences in TCM constitution between groups, so as to examine the TCM constitution closely related to the disease. As advised, a case-control study or prospective cohort study should be carried out for the possible high-risk TCM constitution of a disease. This is to identify the causal relationship between the TCM constitution and the disease, especially true for heavy chronic disease like stroke. For a thorough analysis of the factors influencing TCM constitution, interference factors shall be controlled in line with the international norms of observational research methodology of quality evaluation. This is to guarantee the accuracy of TCM constitution identification, and collect demographic disease information in detail. STROBE statement is recommended to make standardized reports when writing research papers [55], and thereby improving research quality and providing high-quality original evidence for systematic evaluation.

## 5. Conclusions

The meta-analysis of 11,211 subjects finds that PDC, QDC, and BSC types are the main TCM constitutions in IS patients, QDC and PDC are risk factors for IS, and QDC is most closely related to IS. It is suggested that the future clinical observation research on the correlation between TCM constitutions and diseases should improve research quality, comprehensively explore the factors influencing TCM constitution, and thoroughly study the association between constitution and disease. Thereby, it could provide valuable reference for the clinical diagnosis and treatment of differentiated syndrome and diseases and adopt relevant implementation and reporting norms of evidence-based medicine.

## Figures and Tables

**Table 1:** The basic characteristics and quality score of studies included.

Study ID	Study type	Area	Participant	Sample size	Age	Gender ratio (M/F)	Constitution	Quality score
Lijin [13]	CS	Guangdong	Hospital	183	67.8 ± 11.2	107/76	9 types	6
Sunshuai [14]	CS	Beijing	Hospital	61	64.13 ± 7.525	45/16	9 types	8
Zhangxiu [15]	CS	Fujian	Hospital	150	70.4 ± 11.2	87/63	9 types	6
Liuxia [16]	CS	Beijing	Hospital	125	65.69 ± 11.54	79/46	8 types (without Balanced)	5
Luyong [17]	CS	Shanghai	Hospital	200	70.64 ± 4.33	102/98	8 types (without inherited special)	7
Mawen [18]	CS	Shandong	Hospital	98	NI	53/55	9 types	5
Songwei [19]	CS	Shandong	Hospital	127	69 ± 18	78/49	9 types	6
Lixiu [20]	CS	Shandong	Hospital	523	66.87 ± 10.26	325/198	9 types	9
Liyu [21]	CS	Beijing	Hospital	227	73.51 ± 8.19	100/78	9 types	7
Liangmin [22]	CS	Guangdong	Hospital	150	68.08 ± 12.15	83/67	9 types	8
Maxian [23]	CS	Jiangsu	Hospital	299	NI	178/121	9 types	8
Wuhong [24]	CS	Shandong	Hospital	403	NI	254/149	9 types	6
Xuyin [25]	CS	Beijing	Hospital	500	62.3 ± 11.9	325/175	9 types	8
Zhenxiao [26]	CS	Shanghai	Community & hospital	475	NI	232/243	Phlegm-dampness	8
Jiaai [27]	CC	Liaoning	Hospital	510	NI	261/249	9 types	7
Lili [28]	CS	Guangdong	Hospital	276	65.95 ± 11.03	187/89	9 types	9
Zhuangshan [29]	CS	Shandong	Hospital	210	64.43 ± 9.58	114/96	9 types	8
Jiaai [30]	CS	Liaoning	Hospital	620	NI	315/305	9 types	9
Zhumei [31]	CS	Guangxi	Hospital	90	68	53/37	9 types	8
Wangkai [32]	CS	Hubei	Hospital	156	72.56 ± 8.12	40/38	9 types	9
Wangqun [33]	CS	Shandong	Hospital	142	67.52 ± 10.62	78/64	9 types	7
Lizheng [34]	CS	Henan	Hospital	110	59.45 ± 11.44	64/46	9 types	8
Zhanghai [35]	Cohort	Shanghai	Hospital	168	70.14 ± 11.4	101/67	9 types	8
Liangkun [36]	CS	Shandong	Hospital	100	NI	NI	9 types	6
Mengshu [38]	Cohort	Guangdong	Hospital	803	NI	531/272	9 types	9
Fengpeng [39]	CS	Jilin	Hospital	202	64.23 ± 10.04	116/86	9 types	7
Cuisai [41]	CS	Shandong	Hospital	153	64.05 ± 11.84	99/54	9 types	8
Huangling [42]	CS	Jiangxi	Hospital	180	66.33 ± 9.28	105/75	9 types	7
Fuxin [43]	CS	Jilin	Hospital	176	63.56 ± 10.41	112/64	9 types	6
Lili [47]	CS	Jilin	Hospital	248	63.40 ± 10.38	166/82	9 types	7
Jichuan [49]	CS	Guangdong	Hospital	100	49.61 ± 4.85	61/39	9 types	6
Gaocheng [52]	CS	Shandong	Hospital	62	NI	36/26	9 types	6
Gaoyang [53]	CS	Beijing, Guangdong, Henan, Jilin, Shanxi	Hospital	1741	62.7 ± 9.63	1106/635	9 types	7
Wanghua [37]	CS	Hebei	Hospital	198	NI	126/72	9 types	8
Xieqing [40]	CS	Yunnan	Hospital	52	NI	40/12	9 types	8
Zouzhi [44]	CC	Guangdong	Hospital	40	71.27 ± 15.34	18/22	9 types	4
Zhouhan [45]	CS	Hunan	Hospital	303	64.87 ± 11.19	175/128	9 types	9
Wangfang [46]	Cohort	Beijing, Shanxi	Hospital	274	62.49 ± 8.63	188/86	9 types	7
Wuxiao [48]	CS	Beijing	Community	400	NI	249/151	9 types	9
Liu et al. [50]	CS	Shanghai	Community	124	69.33 ± 7.53	66/58	9 types	8
Yuhong [51]	CS	Shandong	Hospital	252	65.12 ± 12.30	145/107	9 types	8

CS: cross-sectional study; CC: case-control study; NI: no information.

**Table 2:** Meta-analysis of other TCM constitutions' distribution in IS and the general population.

TCM constitution	Event/total number		Heterogeneity test		OR	95% CI	*Z*	*P*
	IS population	General population	*I* ^2^ (%)	*P*				
Blood stasis constitution (BSC)	93/620	105/3988	5	0.38	1.44	0.91–2.27	1.57	0.12
Yang-deficiency constitution (YADC)	82/542	883/3910	79	0.002	1.29	0.56–3.00	0.60	0.55
Yin-deficiency constitution (YIDC)	88/542	352/3910	41	0.17	1.19	0.69–2.05	0.63	0.53
Damp-heat constitution (DHC)	75/542	1218/3910	66	0.03	0.87	0.39–1.92	0.35	0.73
Qi stagnation constitution (QSC)	21/542	64/3910	16	0.31	1.01	0.42–2.42	0.01	0.99
Balance constitution (BC)	110/542	1311/3910	95	<0.00001	0.37	0.10–1.38	1.48	0.14
Inherited special constitution (ISC)	3/420	85/3788	0	0.78	0.53	0.16–1.71	1.06	0.29

**Table 3:** Meta-analysis of rate of other TCM constitutions in IS patients.

Constitution type	Studies included	Proportion (%)	95% CI
Yin deficiency	40	13	[0.11, 0.15]
Yang deficiency	40	10	[0.08, 0.12]
Balance	40	7	[0.05, 0.11]
Qi stagnation	40	7	[0.05, 0.09]
Damp-heat	40	6	[0.05, 0.07]
Inherited	40	1	[0.01, 0.02]
